# Sir James Porter's Record as Medical Director-General

**Published:** 1913-05-10

**Authors:** 


### THE NAVY HOSPITAL SERVICE.

### Sir James Porter's Record as Medical Director-General.

The retirement of Surgeon-General Sir James Porter, K.C.B., from the post of Medical Director-General of the Navy, which was recently announced in The Hospital, provides an instructive point from which to view the recent progress of the Service, to summarise the changes made, and to indicate by a review of these what are likely to be the lines of future developments.

### Discontent in 1908.

Sir James Porter was appointed Director-General of the Navy in 1908, and it is a matter of common knowledge that at that time there was a considerable amount of discontent in all grades, both commissioned and non-commissioned. The changes that have taken place in the past five years indicate the grounds of that discontent, which they have begun to remedy, though the plans in which Sir James Porter has been most interested, and for which he has worked extremely hard, are far from being completely realised. The pay was one main cause of dissatisfaction, and the Durnford Committee, which was appointed in 1908, recommended an increase in pay for the medical officers. The demand for an increase in fact has been granted to some extent, though it is, of course, still a question as to whether the pay is sufficient for the duties and state of efficiency which are demanded. Another great change has been the institution of the medical school at Greenwich, which has now been working for about two years. This was established mainly for three objects: For the giving of instruction to acting surgeons on entry to the Service; for the post-graduate instruction of surgeons before their promotion to the rank of staff surgeons; and, thirdly, for the post-graduate instruction of senior medical officers—that is to say, of men with fourteen years' seniority. The school is affiliated to the *Dreadnought* Hospital, Greenwich, at which the clinical instruction is carried out.

### Changes in Salaries and Staff.

There have been also administrative changes. Here there is again an increase in pay, notably for the sisters, and also a new appointment—namely, the creation of the post of superintending sister, between that of sister and head-sister. Perhaps institutional readers outside the Service may be reminded that the nursing staff consists of what is known as the Sick Berth Staff, and as it is, of course, impossible to have women on a man-of-war, the nurses are male nurses, with sisters over them, who have certain teaching responsibilities, and this arrangement obtains throughout the Service abroad and at home, the male nurses being in regular order drafted from the ships to the naval hospitals, of which, by the way, the number is considerably larger than the public is aware. This arrangement of drafting out and home was one of the causes which led to the creation of the new post of superintending sister. For a woman who had attained the rank of acting head-sister at a naval hospital abroad did not like to fall back to the rank of sister, as she used to have to do on her return home.

By the new arrangement the sister who is placed in the responsible position abroad becomes a superintending sister, and that intermediate rank, and the higher pay which goes with it, she retains on her return. Then, again, important changes have been made in regard to the pay and duties of the male nurses. Their system of education has been revised, and their courses of instruction have been both lengthened and enlarged in scope. For instance, the special subjects of bacteriology and operative surgery have been added, and special pay is given as a result of proficiency in these. In addition to this special pay is also given for those men to whom special duties are allotted; those, for instance, who undertake dispensing, or serve in the infectious wards or the mental wards. A system of examinations has also been instituted for the grade of chief sick berth steward.

### New Drug-making Department.

The whole Navy Medical Service is, of course, centralised under the Medical Director-General, who is thus able to insist on and carry out great changes on uniform lines throughout the whole Service: a point, of course, which at once differentiates the Navy hospitals from those of the voluntary system, or even, for that matter, from the Poor-Law infirmaries under the Local Government Board. Sir James Porter took advantage of the power residing in his office to have the diet schemes prevailing in the Medical Service revised throughout, and also to effect important changes in the stores department, the effect of which has been to obviate a great deal of waste and inefficiency. Another very great change has been the installation of machinery for the manufacture of many of the drugs required in the Service. Many medicines and tabellæ are made by this machinery, which ensures a standard quality, and has proved much cheaper in the long run.

Previously all the drugs required were purchased by contract. Now that system, which has the apparent advantage of simplicity, must, of course, give a profit to the makers who accept it, and the Royal Commission on Proprietary Medicines has helped to reveal how large the profits on such medicines are and how greatly the actual cost of the ingredients is raised by the large sums necessarily spent in advertising them. Once the capital expenditure of installing the requisite machinery has been incurred, there is no further expense to be provided for beyond that of upkeep. Since analysis has made secrecy of compounding practically impossible, everyone knows at how small a price emulsions and such compounds can be made, and the Service is now reaping the profit which used to go to the contracting firms. The drug-making department is an immense concern. There are the great naval hospitals of Chatham, Plymouth, and Haslar, all the marine infirmaries, the sick quarters at Osborne and Dartmouth, and the Medical Service connected with the Dockyards, where alone 50,000 men are employed. That is the "public" which the drug department of the Navy Medical Service has to supply. All the Navy dispensers are fully qualified pharmaceutical chemists, and Sir James Porter has tried hard to increase their status and their pay.

### Difficulties of Naval Medical Officers.

The Sick Berth Staff—that is, the male nurses—who are mainly recruited from the clerk class, is practically sufficient in numbers; but the same thing cannot be said of the Navy medical officers. The commissioned ranks of the Navy Medical Service have many difficulties to contend with. It is suspected by the profession as a whole, which, like the general public, is very ignorant of the actual conditions, opportunities, and limitations of the Service. Taking the last first, one may sum up the disadvantages in the sentence : Naval medical officers have responsibility without authority. But while they are responsible for the medical treatment of the sick, which involves, of course, the adequate carrying out of their instructions, they have no power over the Sick Berth Staff, except to report them to the Executive. The delays and misunderstandings inevitable with such a system are extremely galling to the medical officers, who may virtually be laughed at by their subordinates, seeing that these well know how clumsy and slow working is the existing machinery for bringing them to book in case of indiscipline or misbehaviour. The advantages are seen in the magnificently equipped and organised Hospital Service which the Navy possesses.

Voluntary and Naval Hospitals Compared.

It is perhaps a pardonable prejudice on the part of naval medical officers to affirm that the voluntary hospitals cannot compare with the naval hospitals. All the conditions, they say, are in favour of the latter, although, of course, such special departments—as that of the Finsen Light—do not exist in a naval hospital. It does not exist because it is not required. But in other respects, in construction, in ward equipment, in diet, in co-ordination, the naval hospitals are unrivalled, though laymen will not believe it, because they never visit them. In naval hospitals, in the first place, there is no charity, which means in practice that every person, no matter what his illness is, is entitled to treatment. A man cannot be rejected because his disease is infectious, nor can another institution be suggested in a septic case. The naval hospital must provide for everything, and have all classes of cases in the separate accommodation provided for them. Haslar is held up by naval men as a fine example of hospital construction, and has been brought up to date. The subject of hospital administration is seen in its most comprehensive aspects at a naval hospital.

There are fewer vacancies in the Army and Indian Medical Services. The men are better treated there. Recognition for services is more liberal than in the Navy, where the Executive appear to have established a prescriptive right to all the honours. Sir James Porter has tried hard to remedy this, and to obtain a more equitable adjustment. The evils of the present system are very obvious. The commissioned officers get discontented because much good work and distinction goes without recognition, and the Sick Berth Staff, being associated with the medical officers, and yet controlled, not by them, but by the Executive, do not know to whom to look for advancement. Something, however, has been achieved under Sir James Porter's directorship. Pensions for the widows of officers have existed for some time, but these are now being extended to the widows of the men whose right to them is recognised.

Hospital Ships for the Navy.

Sir James Porter has worked very hard to obtain a hospital ship for the Navy, and his efforts have been crowned at last with success. Probably one of the reasons why he has insisted so much on its importance is that he has seen more active service than any other naval officer, and the experience gained during war concerned the landing of naval brigades and shore work generally. The function of a hospital ship is somewhat different. It is to follow the fleet and to receive the acute cases from the men-of-war, and remove them to the nearest naval hospital. This may sound a simple procedure, but in reality it is a very obscure one. Nothing is known about the transfer of patients from a man-of-war to a hospital ship, and this evidentiy is a very complicated function, and should be practised as far as possible in time of peace.

The hospital ship may certainly be made a great blessing, but its functions require to be better understood than naval peace allows them to be. This really affords the conclusion of the whole matter. Nobody in the British Navy knows or can know much about war at sea, because there has not been one for so long. Should a naval war occur it would achieve by painful experience at a stroke many of the reforms for which Sir James Porter has struggled, and many of the administrative changes for which he has struggled in vain. In war you cannot have responsibility without authority. But only a war, it seems, will make this fact recognised. The avoidable suffering that will take place will drive home this lesson, as it was driven home in the Army, for instance, by the Boer War. In the meantime the Medical Director-General of the Navy has to work quietly and persistently through as many worries and checks as the lower officers towards those reforms which the whole Service knows to be required. This is what Sir James Porter has done, and it has made his directorship of five years a memorable and progressive one. His successor, Surgeon-General May, C.B., will, no doubt, work for an extension of the reforms already made.

